# Role of C9orf72 hexanucleotide repeat expansions in ALS/FTD pathogenesis

**DOI:** 10.3389/fnmol.2024.1322720

**Published:** 2024-01-22

**Authors:** Yanyan Geng, Qixu Cai

**Affiliations:** ^1^Clinical Research Institute of the First Affiliated Hospital of Xiamen University, Fujian Key Laboratory of Brain Tumors Diagnosis and Precision Treatment, Xiamen Key Laboratory of Brain Center, the First Affiliated Hospital of Xiamen University, School of Medicine, Xiamen University, Xiamen, Fujian, China; ^2^State Key Laboratory of Vaccines for Infectious Diseases, School of Public Health, Xiamen University, Xiamen, Fujian, China

**Keywords:** C9orf72, hexanucleotide repeat expansions, G-quadruplex, phase separation, amyotrophic lateral sclerosis, frontotemporal dementia, neurodegenerative diseases

## Abstract

Amyotrophic lateral sclerosis (ALS) and frontotemporal dementia (FTD) are progressive neurological disorders that share neurodegenerative pathways and features. The most prevalent genetic causes of ALS/FTD is the GGGGCC hexanucleotide repeat expansions in the first intron region of the chromosome 9 open reading frame 72 (C9orf72) gene. In this review, we comprehensively summarize the accumulating evidences elucidating the pathogenic mechanism associated with hexanucleotide repeat expansions in ALS/FTD. These mechanisms encompass the structural polymorphism of DNA and transcribed RNA, the formation of RNA foci via phase separation, and the cytoplasmic accumulation and toxicities of dipeptide-repeat proteins. Additionally, the formation of G-quadruplex structures significantly impairs the expression and normal function of the C9orf72 protein. We also discuss the sequestration of specific RNA binding proteins by GGGGCC RNA, which further contributes to the toxicity of C9orf72 hexanucleotide repeat expansions. The deeper understanding of the pathogenic mechanism of hexanucleotide repeat expansions in ALS/FTD provides multiple potential drug targets for these devastating diseases.

## 1 Introduction

**FIGURE 1:**
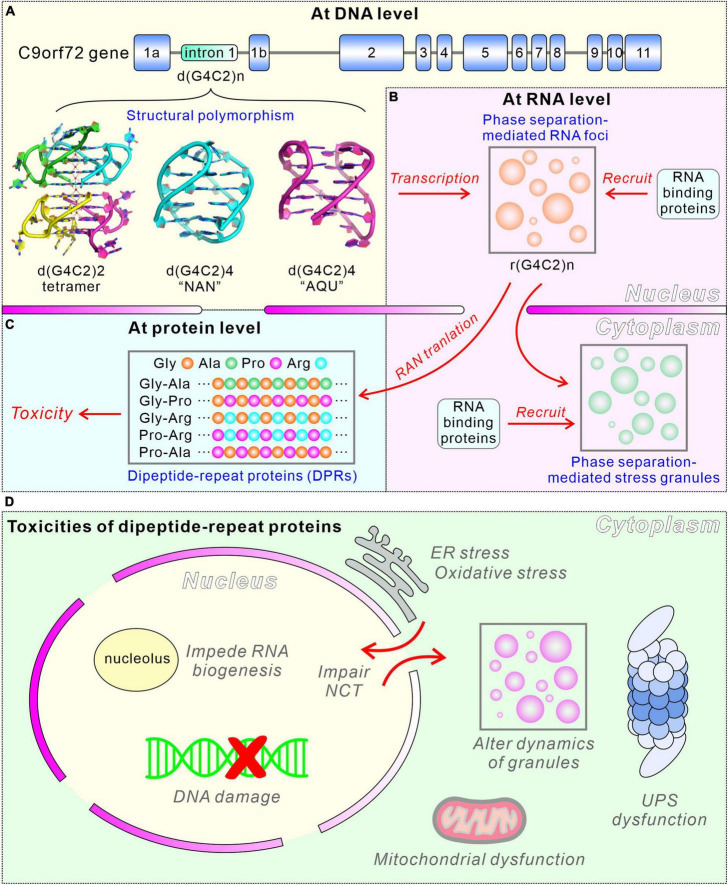
Pathogenic mechanisms of C9orf72 hexanucleotide repeat expansions in ALS/FTD. **(A)** Structural polymorphism of G4C2 repeat expansions of DNA. **(B)** Formation of RNA foci via phase separation. **(C)** Multiple pathogenic mechanisms of G4C2 repeat expansions at protein level. **(D)** Schematic diagrams showing the toxicities of dipeptide-repeat proteins.

Amyotrophic lateral sclerosis (ALS) is a neurological disorder mainly characterized by the degeneration of motor neurons, leading to symptoms including muscle weakness, muscle atrophy and ultimately death from respiratory failure (Rowland and Shneider, 2001), while frontotemporal dementia (FTD) refers to a broad spectrum of neurological disorders marked by progressive damage to the temporal and frontal lobes of the brain (Graff-Radford and Woodruff, 2007; Rademakers et al., 2012). Presently, both ALS and FTD are recognized as part of a broad neurodegenerative continuum that shares common neurodegenerative pathways and features. This includes the presence of TAR DNA-binding protein 43 (TDP-43) cytoplasmic inclusions within the central nervous system (Neumann et al., 2006; Lillo and Hodges, 2009), which was considered as a potential biomarker for ALS/FTD (Majumder et al., 2018). Furthermore, both ALS and FTD were genetically linked to the expanded GGGGCC (G4C2) hexanucleotide repeats in the first intron region of the chromosome 9 open reading frame 72 (C9orf72) gene (Figure 1A). This genetic abnormality was identified as the most common genetic cause of familial ALS and familial FTD (DeJesus-Hernandez et al., 2011; Renton et al., 2011; Majounie et al., 2012).

Tremendous progress and hypothesis have greatly contributed to our understanding of the pathologic mechanism of C9orf72 hexanucleotide repeat expansions in ALS/FTD (Edbauer and Haass, 2016; Gitler and Tsuiji, 2016; Haeusler et al., 2016; Goodman et al., 2019). Both the C9orf72 hexanucleotide repeat expansions and the transcribed RNA, were reported to adopt polymorphic unusual secondary structures, such as G-quadruplexes and hairpin, which were involved in the ALS/FTD pathogenesis via several potential mechanisms (Fratta et al., 2012; Reddy et al., 2013; Haeusler et al., 2014). Moreover, the non-ATG translation of the hexanucleotide repeat expansions leads to the accumulated production of cytoplasmic dipeptide-repeat (DPR) proteins, whose toxicity is also considered as a contributing factor to the pathology of diseases (Ash et al., 2013; Gendron et al., 2013; Mackenzie et al., 2013; Mori et al., 2013a,c). These findings represent crucial steps in unraveling the complex pathogenic mechanisms associated with C9orf72 hexanucleotide repeat expansions in ALS/FTD.

In this mini review, we focus on the pivotal role played by C9orf72 hexanucleotide repeat expansions in the pathogenesis of ALS/FTD across multiple dimensions, as visually represented in Figure 1. We present a comprehensive summary of recent progress, highlighting the structural polymorphism observed in both the DNA and transcribed RNA of C9orf72 hexanucleotide repeat expansions. Additionally, we explore the mechanism of the formation of RNA foci via phase separation, elucidating its profound implications for the pathogenesis of ALS/FTD. Moreover, we delve into the adverse effects of G-quadruplex formation, which significantly impairs the expression and normal function of C9orf72 protein. Meanwhile, we discuss the cytoplasmic accumulation and toxicity of DPR proteins, as well as the role of sequestered and enriched RNA binding proteins (RBPs) by G4C2 RNA, all of which contribute substantially to the overall toxicity related to hexanucleotide repeat expansions in ALS/FTD.

## 2 Structural polymorphism of G4C2 repeat expansions of DNA

G-quadruplex refers to a unique four-stranded helical structure which is formed by guanosine-rich DNA or RNA sequences (Sen and Gilbert, 1988). The fundamental structural unit of a G-quadruplex, known as a G-tetrad, is composed of four guanine molecules arranged in a cyclic Hoogsteen hydrogen-bonded square planar structure (Sannohe and Sugiyama, 2010). Two or more G-tetrads stack to form a G-quadruplex, which is stabilized by monovalent cations (Huppert, 2008). In the context of ALS/FTD, it was reported that the guanosine-rich C9orf72 G4C2 hexanucleotide repeat expansions can fold into polymorphic G-quadruplex *in vitro* (Haeusler et al., 2014; Brcic and Plavec, 2015, 2018; Sket et al., 2015; Zamiri et al., 2015; Zhou et al., 2015, 2018; Figure 1A). Notably, the highly variable size of the hexanucleotide repeat expansions (DeJesus-Hernandez et al., 2011; Renton et al., 2011; Haeusler et al., 2016), as well as the complexity of the G-quadruplex formed by G4C2 repeat sequence, give rise to a dramatic structural diversity of G-quadruplex structures folded by the C9orf72 G4C2 repeat expansions (Haeusler et al., 2014; Zhou et al., 2015, 2018).

In recent years, various biophysical methods have been employed to elucidate the folding and structures of G4C2 repeat expansions with varying length, highlighting the structural polymorphism of G4C2 repeat expansions and the potential existence of multiple architectures. Using circular dichroism (CD) and nuclear magnetic resonance (NMR) spectroscopy, Zhou et al. (2015) investigated the structural heterogeneity of two, three, four and five repeats of G4C2 DNA (referred to as d(G4C2)2, d(G4C2)3, d(G4C2)4 and d(G4C2)4, respectively). They further employed NMR to determine the topology of the G-quadruplex formed by d(G4C2)4, revealing a monomeric chair-type G-quadruplex with a four-layer antiparallel G-tetra core and three edgewise loops (Zhou et al., 2015).

Brcic and Plavec (2015) carried out an *in vitro* investigation involving several mutational variants of d(G4C2)4 oligonucleotides. Notably, they observed that d[(G4C2)3G4] could adopt two predominant structures with antiparallel topologies (Brcic and Plavec, 2015). To enhance structural stability, they introduced a substitution of dG21 with 8-bromoguanine (8Br-dG), a residue that favors a syn glycosidic conformation (Sarma et al., 1974). Interestingly, they found that the folding conditions, including solution pH and the rate of cooling, exert a substantial influence on the structure. They solved the structure of d[(G4C2)3G4] formed in a neutral pH solution with slow cooling (referred to as NAN for neutral and annealing) (Brcic and Plavec, 2015), as well as an alternative structure formed in an acidic pH solution with fast cooling (termed AQU for acidic and quenching) using NMR technique (Brcic and Plavec, 2018). These investigations collectively demonstrate the structural polymorphism of G-quadruplex formed by G4C2 repeat expansions (Figure 1A).

Due to the structural diversity inherent in G-quadruplex formed by G4C2 repeat expansions, Zhou et al. (2018) employed biochemical purification methods to isolate and prepare these G-quadruplex *in vitro*. Initial screening using CD and NMR methods revealed that d(G4C2)2 could form mixed parallel and antiparallel topologies (Zhou et al., 2015). Subsequently, using anion exchange chromatography, they successfully separated different conformations of d(G4C2)2. The various purified fractions were subjected to CD melting experiments and NMR spectroscopy, revealing distinct properties of each fractions (Zhou et al., 2018). Notably, one of the major fractions, designated as the F5 fraction, was successfully crystalized in a K^+^ solution (Zhou et al., 2018). To solve the phase problem, d(G4C2)2 was annealed in Ba^2+^ solution, leading to the crystallization of d(G4C2)2-Ba. With the help of the anomalous signal of Ba^2+^, the structure of d(G4C2)2-Ba was solved and subsequently used as the template to solve the structure of d(G4C2)2-K by molecular replacement method. The structure revealed that d(G4C2)2 folds into a novel eight-layer parallel tetrameric G-quadruplex (Geng et al., 2021). Interestingly, the crystal structure displayed two distinct forms of tetrameric G-quadruplexes, referred to as Form-1/7 (where the dG1 base in one dimeric block stacks with the dG7 base in the opposite dimeric block) and Form-1/1 (where the dG1 base in one dimeric block stacks with the dG1 base in the opposite dimeric block) respectively. These novel multimeric G-quadruplex structures formed by d(G4C2)2 underscore the complexity and potential for oligomerization of G-quadruplexes generated by G4C2 repeat expansions (Figure 1A).

In order to comprehend the functional consequence and pathological implications of the structural polymorphism of G4C2 repeat expansions, Haeusler et al. (2014) examined the transcription of the G4C2 repeat expansions using an *in vitro* transcription assay. Their findings unveiled a repeat-length-dependent accumulation of transcripts that were prematurely terminated within the repeat expansions. The abortive transcription was also characterized in patient cells with C9orf72 G4C2 repeat expansions. Moreover, they identified an essential nucleolar protein, nucleolin, which specifically binds to these transcripts. This interaction led to impaired nucleolar function and nucleolar stress in patient cells, thus it is considered as one of the pathogenic mechanisms underlying C9orf72-linked ALS/FTD (Haeusler et al., 2014) (see below). Nevertheless, it is still unclear how the diverse structures formed by G4C2 repeat expansions impact the specific pathogenic mechanisms.

## 3 Formation of RNA foci via phase separation

It has been proved that the transcribed RNA from G4C2 repeat expansions [termed r(G4C2)n] can indeed form various structures, including G-quadruplexes (Fratta et al., 2012; Reddy et al., 2013; Haeusler et al., 2014), hairpin structures (Su et al., 2014) and duplex structures (Maity et al., 2021). These diverse structures have been confirmed through topological investigations using gel-shift assay, CD spectroscopy and NMR spectroscopy. Furthermore, it has been observed that the equilibrium between G-quadruplex, hairpin structure and duplex structure was regulated by factors like pH and temperature (Bozic et al., 2020). This phenomenon is reminiscent of the distinct G-quadruplex structures formed by d[(G4C2)3G4], as previously discussed, influenced by pH and rate of cooling (Brcic and Plavec, 2015, 2018). However, it’s important to note that the detailed molecular structure of G-quadruplex formed by r(G4C2)n has not been resolved yet.

The accumulation of RNA foci in the nucleus, formed by transcripts containing the G4C2 repeat expansions, is a recognized pathological hallmark shared by ALS/FTD. Phase separation of biological macromolecules have been extensively studied, and plays a crucial role in the formation and regulation of membraneless organelles (Banani et al., 2017; Shin and Brangwynne, 2017; Wu et al., 2020; Roden and Gladfelter, 2021), particularly in the neurodegenerative diseases (Zbinden et al., 2020). Jain and Vale (2017) made a significant discovery by observing that G4C2 repeat expansions undergo a solution-gel phase separation *in vitro* at a critical repeat number similar to what is observed in ALS/FTD patients. The G4C2 repeat-containing RNA can form RNA foci via phase separation in human cells, suggesting that the gelation of G4C2 repeat-containing RNA contributes to the pathology of ALS/FTD disease via phase separation (Fay et al., 2017; Jain and Vale, 2017; Figure 1B).

Phase separation of biological macromolecules depends on the multivalent intermolecular interactions (Banani et al., 2017; Shin and Brangwynne, 2017). Given the tetrameric structure of G-quadruplex formed by d(G4C2)2 (Geng et al., 2021), it is reasonable to speculate that G-quadruplex formed by r(G4C2)2 is also multimeric. The multimeric organization provides the specific multivalent intermolecular interactions, but not the weak and unspecific interactions between RNA, necessary for the phase separation of G4C2 repeat-containing RNA (Shen et al., 2023). Furthermore, the phase separation of RNA occurs at a boundary condition of increasing effective valence contributed by the increasing repeat number and specific intermolecular interaction. This could explain why ALS/FTD disease is triggered after the G4C2 repeat expansions reach a certain threshold of repeat number (Haeusler et al., 2016; Jain and Vale, 2017). Additionally, the highly enriched condensates of r(G4C2)n can further recruit RNA-binding proteins through phase separation. This contributes to the formation of RNA granules, including the nuclear RNA foci and cytoplasmic stress granules, all of which play a role in the pathogenesis of the diseases (Fay et al., 2017; Figure 1B).

## 4 Multiple pathogenic mechanisms of G4C2 repeat expansions at protein level

### 4.1 Loss-of-function pathogenic mechanisms

The C9orf72 protein interacts with Smith-Magenis syndrome chromosome region candidate gene 8 (SMCR8) and WD repeat-containing protein 41 (WDR41) (Amick et al., 2016; Sullivan et al., 2016), forming a stable ARF GTPase-activating protein (GAP) complex (Su et al., 2020, 2021; Tang et al., 2020; Norpel et al., 2021). This ARF GAP complex was reported to be involved in regulation of autophagy (Amick et al., 2016; Sullivan et al., 2016; Yang et al., 2016), and is critical for microglial function (O’Rourke et al., 2016) and the modulation of actin dynamic in motor neurons (Sivadasan et al., 2016). The unusual secondary structures formed by the G4C2 repeat expansions in the first intron region of C9orf72 gene significantly reduce the expression of C9orf72 protein by the abortive transcription (DeJesus-Hernandez et al., 2011; Renton et al., 2011; Haeusler et al., 2014; Shi et al., 2018). Additionally, the epigenetic mechanisms, including the methylation of cytosine and histone, also contribute to the reduction of C9orf72 expression (Belzil et al., 2013; Xi et al., 2014). For the human induced motor neurons, the exogenous restoration of C9orf72 expression can rescue the survival of neurons (Shi et al., 2018), suggesting that the decrease of C9orf72 protein contributes to the pathogenesis of ALS/FTD. However, the C9orf72 loss-of-function mouse model studies have found that the knock down or knockout of C9orf72 gene does not recapitulate ALS/FTD (Lagier-Tourenne et al., 2013; Koppers et al., 2015; Atanasio et al., 2016; Burberry et al., 2016; Jiang et al., 2016; O’Rourke et al., 2016; Sudria-Lopez et al., 2016; Sullivan et al., 2016; Ugolino et al., 2016; Liu et al., 2022), although some models showed mild motor or cognitive phonotypes or reduced survival (Table 1; Atanasio et al., 2016; Burberry et al., 2016; Jiang et al., 2016; Sudria-Lopez et al., 2016; Ugolino et al., 2016; Batra and Lee, 2017; Balendra and Isaacs, 2018; Liu et al., 2022). This indicates that C9orf72 loss of function is not sufficient to lead to ALS/FTD, further highlighting the complex nature of these neurodegenerative diseases.

**TABLE 1:** C9orf72 mouse models.

	Methods	Phenotypes
Loss of function	C9orf72 depletion in the adult mouse CNS by ASO injection (Lagier-Tourenne et al., 2013)	Normal
	Neural-specific ablation of C9orf72 (Koppers et al., 2015)	Reduced body weight; Normal motor function.
	Remove exons 2-6 of C9orf72 (Jiang et al., 2016)	Reduced body weight; Splenomegaly and enlarged lymph nodes; Decreased hemoglobin and packed cell volume; Decreased percentage of lymphocytes; Increased percentage of neutrophils in blood; Mild social interaction and social recognition abnormalities; Mild motor deficits.
	Knockout of C9orf72 full gene (Atanasio et al., 2016)	Mild motor deficits; Lymphadenopathy and splenomegaly; Mixed inflammatory infiltrates in multiple organs; Systemic lupus erythematosus-like disease; Reduced survival.
	Model 1: Remove exons 2-6 of C9orf72 Model 2: ZFN-mediated knockout of C9orf72 (O’Rourke et al., 2016)	Progressive splenomegaly and lymphadenopathy; Age-related neuroinflammation
	Full ablation of C9orf72 (Sudria-Lopez et al., 2016)	Reduced survival; Reduced body weight; Normal motor function; Enlarged lymph nodes and splenomegaly.
	CRISPR/Cas9-mediated knockout of C9orf72 (Sullivan et al., 2016)	No growth defects; Lymph node and spleen enlargement.
	Model 1: Remove exons 2-6 of C9orf72 Model 2: Remove exons 2-6 of C9orf72 without selection cassette Model 3: CRISPR/Cas9-mediated knockout of C9orf72 (Burberry et al., 2016)	Splenomegaly; Neutrophilia; Thrombocytopenia; High mortality rate.
	Remove exons 2-6 of C9orf72 without selection cassette (Ugolino et al., 2016)	Decreased life span; Splenomegaly; No obvious neuronal cell death in brain or spinal cord.
	Jackson Lab Stock No. 027068 (O’Rourke et al., 2016): ZFN-mediated knockout of C9orf72 (Liu et al., 2022)	Motor deficits; Hyperactivity of Purkinje cells; Enhanced BK channel in the cerebellum.
Gain of function	Expression of (G4C2)66 throughout CNS by AAV-mediated somatic brain transgenesis (Chew et al., 2015)	Nuclear RNA foci; Inclusions of poly(GP), poly(GA) and poly(GR) DPR inclusions; TDP-43 pathology; Cortical neuron and cerebellar Purkinje cell loss; Astrogliosis; Reduced body weight; Behavioral abnormalities.
	BAC containing exons 1-6 of human C9orf72 gene with ∼500 repeats of G4C2 motif (Peters et al., 2015)	Sense and antisense RNA foci; Poly(GP) DPR inclusions; No TDP-43 pathology; Survival, motor and cognitive systems are normal.
	BAC containing human C9orf72 gene with ∼100-1000 repeats of G4C2 motif (O’Rourke et al., 2016)	Widespread RNA foci; Poly(GP) DPR inclusions; Normal behavior; No neurodegeneration.
	BAC containing human C9orf72 gene with up to 500 repeats of G4C2 motif (Liu et al., 2016)	Decreased survival; Kyphosis, reduced activity, hyperactivity when provoked, clasping and intermittent seizures; Motor neuron disease; Neurodegenerative changes; Sense and antisense RNA foci; Poly(GA) and poly(GP) aggregates; Nuclear and cytoplasmic TDP-43 aggregates.
	BAC containing exons 1-5 of human C9orf72 gene with ∼110 or ∼450 repeats of G4C2 motif (Jiang et al., 2016)	Sense and antisense RNA foci; Cytoplasmic poly(GP), poly(GR) and poly(GA) aggregates; Age-dependent cognitive impairment and anxiety-like behavior; No TDP-43 mislocalization or aggregation with increased phosphorylated TDP-43.
	Expression of GFP-(GA)50 or GFP-(GA)50-mut in the CNS by AAV-mediated somatic brain transgenesis (Zhang et al., 2016)	Poly(GA) aggregation; Brain atrophy and neurotoxicity; Hyperactivity, anxiety-like behavior, motor and cognitive deficits; rare phosphorylated TDP-43 inclusions;
	Expression of (GA)149, 31 amino acids corresponding to the 3′ region of the poly(GA) reading frame in patients and a C-terminal CFP tag (Schludi et al., 2017)	Poly(GA) inclusions; Mild TDP-43 phosphorylation; No TDP-43 inclusions and mislocalization; Microglia activation without astrogliosis; Progressive motor deficits.
	Expression of 10 pure or 102 interrupted G4C2 repeats in the brain by AAV-mediated somatic brain transgenesis (Herranz-Martin et al., 2017)	RNA foci; Extensive DPR pathology; Disease-related NMJ pathology; Progressive gait and behavioral deficits.
	Conditional expression of GFP-(PR)28 in neurons (Hao et al., 2019)	Poly(PR) aggregation; No TDP-43 inclusions; Decreased survival; Smaller body size; Smaller brain volumes; Motor deficits, motor-related neurodegeneration and gliosis for heterozygous mice; No cytoplasmic TDp-43 inclusions.
	Knockin of 80 G4C2 repeats with human flanking fragments within exon1a and exon1b at the rat C9orf72 locus (Dong et al., 2020)	Motor deficits; Loss of motor neurons; Hind limb paralysis for females.
	Tet-on inducible expression of 36 × pure G4C2 repeats with 100-bp upstream and downstream human flanking regions (Riemslagh et al., 2021)	Sporadic sense DPR aggregates; No apparent neurodegeneration; No RNA foci; No pTDP-43 pathology; Locomotor phenotype, rapid muscular dystrophy and neuromuscular junction abnormalities.
	Expression of ATG-driven FLAG-(GR)50-eGFP using FAST cassette (Verdone et al., 2022)	No TDP-43 pathology; No reduction in survival; No motor and cognitive impairments; Mild motor neuron loss in males.
	AAV-mediated expression of (G4C2)149 (Jambeau et al., 2022)	Poly(GA), Poly(GR) and Poly(GP) aggregation; Normal survival; Increases in distance traveled, velocity of movement and time spent moving on an open-field assay.

### 4.2 Toxicity of dipeptide-repeat proteins

The transcribed r(G4C2)n can undergo a mechanism called repeat-associated non-ATG dependent translation (RAN translation), leading to the production of dipeptide-repeat (DPR) proteins (Ash et al., 2013; Mori et al., 2013a,c; Zu et al., 2013). This non-ATG initiated translation of the G4C2 repeat expansions from all six potential reading frames can generate five different DPR proteins: Gly-Ala (GA), Gly-Pro (GP) and Gly-Arg (GR) translated by sense (G4C2)n RNA, as well as Pro-Gly (PG) (equivalent to GP), Pro-Arg (PR) and Pro-Ala (PA) translated by antisense (G4C2)n RNA. The accumulation of these unconventionally translated DPR proteins leads to the formation of cytoplasmic insoluble inclusions in neurons, which can be identified by specific DPR antibodies (Ash et al., 2013; Mori et al., 2013c; Zu et al., 2013). Thus, DPR proteins are considered as a pathognomonic feature of ALS/FTD. Especially, the formation of polymorphic RNA G-quadruplex formed by r(G4C2)n can trigger ribosomal frame shifting during translation (Yu et al., 2014), leading to the unconventional translation of DPR proteins.

Two of the Arg-containing DPR proteins, GR and PR, were reported to bind to nucleoli, impede RNA biogenesis and induce cell death, illustrating the toxic effects of DPR proteins (Kwon et al., 2014; Figures 1C, D). Additionally, the Arg-containing DPR proteins directly bind to proteins harboring low complexity domains and impair the assembly, dynamics and function of phase separation-mediated membraneless organelles (Lee et al., 2016; Lin et al., 2016). In iPSC-derived motor neurons, expression of poly(GR) increases oxidative stress and DNA damage, and causes mitochondrial dysfunction (Lopez-Gonzalez et al., 2016). Another DPR, poly(GA), forms ubiquitin/p62-positive inclusions in neuronal cells, indicating the dysfunction of ubiquitin-proteasome system (UPS) leads to the cytotoxicity of DPR proteins (Yamakawa et al., 2015). Poly(GA) also forms abundant inclusions in cells and cerebellar tissue of ALS/FTD patients, and causes impairment of neurite outgrowth, endoplasmic reticulum (ER) stress and neuronal death (Zhang et al., 2014). The mouse model generated by the overexpression of poly(GA) showed the sequestration of proteins involved in proteasomal degradation and nucleocytoplasmic transport (NCT), leading to the neurodegeneration and behavioral abnormalities (Zhang et al., 2016). Overall, these toxic DPR proteins are a key pathological aspect of ALS/FTD, adding to the complexity of these neurodegenerative diseases (Figures 1C, D).

### 4.3 Other downstream pathogenic mechanisms

Subsequently, the transcribed r(G4C2)n may sequester specific proteins, which is a potential mechanism of toxicity associated with hexanucleotide repeat expansions at the protein level. Extensive studies have identified a series of proteins that bind to the transcribed G4C2 repeat expansions through various approaches, including adenosine deaminase RNA-specific B2 (ADARB2), nucleolin, purα, TDP-43 and several heterogeneous nuclear ribonucleoproteins (hnRNPs) (Donnelly et al., 2013; Mori et al., 2013b; Xu et al., 2013; Cooper-Knock et al., 2014; Haeusler et al., 2014). In particular, many of these binding proteins can interact specifically with r(G4C2)n with special secondary structures, such as G-quadruplex and hairpin structures, underscoring the structural specificity of RNA-protein interactions. The interactions may lead to the accumulation of nuclear or cytoplasmic RNA-protein aggregates (Ramaswami et al., 2013) and RNA granules mediated by liquid-liquid phase separation (Ramaswami et al., 2013; Maharana et al., 2018; Hallegger et al., 2021; Yu et al., 2021; Lu et al., 2022), contributing to the toxicity associated with hexanucleotide repeat expansions (Figure 1B).

Using the stable isotope labeling by amino acids in cell culture (SILAC) method, Haeusler et al. (2014) identified nucleolin specifically recognize the RNA G-quadruplex, which was further confirmed by RNA pull down experiments. Nucleolin is a principal component of the nucleolus (Abdelmohsen and Gorospe, 2012), which was found to mislocalize with the G4C2 RNA foci in the neurons of the motor cortex of C9orf72 ALS patients, leading to nucleolar stress and impaired nucleolar function in patient cells. Importantly, treatment of wild type cells with the 21-repeat-containing abortive transcripts recapitulates the nucleolin pathology, indicating that the specific interaction between nucleolin and RNA G-quadruplex is a fundamental determinant of pathogenic mechanism for ALS/FTD (Haeusler et al., 2014).

Another prominent downstream pathogenic mechanism for ALS/FTD is the dysfunctional nucleocytoplasmic transport (Prpar Mihevc et al., 2017). Using a *Drosophila* model-based screening, Zhang et al. (2015) characterized that RNA G-quadruplex formed by r(G4C2)n directly interacts with RanGAP and impairs nuclear import, leading to the nuclear pore pathology in the *Drosophila* model and ALS patient-derived iPSC cells. In the other study, Freibaum et al performed a large-scale genetic screen in a *Drosophila* model, and identified 18 genetic modifiers that encode components of the nuclear pore complex and the protein machinery for nucleocytoplasmic transport, demonstrating the compromised nucleocytoplasmic transport in the *Drosophila* model and C9orf72-linked patient-derived iPSC cells (Freibaum et al., 2015).

### 4.4 C9orf72 gain-of-function mouse models

Besides the C9orf72 loss-of-function mouse models (see above and Table 1), adeno-associated virus (AAV) or bacterial artificial chromosome (BAC)-mediated expression of C9orf72 gene with large G4C2 repeat expansions or DPR proteins was used to generate a number of gain-of-function mouse models (Table 1). With the expression of C9orf72 gene with large G4C2 repeat expansions, most of the mouse model developed both RNA foci and DPR inclusions (Chew et al., 2015; Peters et al., 2015; O’Rourke et al., 2016; Herranz-Martin et al., 2017), while the mouse model with tet-on inducible expression of 36x pure G4C2 repeats only have sporadic sense DPR aggregates, but not RNA foci (Riemslagh et al., 2021). The mouse models with the overexpression of DPR proteins showed extensive DPR pathology, but not RNA foci (Zhang et al., 2016; Schludi et al., 2017; Hao et al., 2019). TDP-43 inclusions were not usually observed in the gain-of-function mouse models. Only two of the mouse models, (G4C2)66 mice and C9-500 mice, displayed TDP-43 inclusion pathology and neurodegenerative phenotypes (Chew et al., 2015; Liu et al., 2016). It was noted that another group reported the absence of survival and motor deficits for C9-500 mice (Mordes et al., 2020; Nguyen et al., 2020a). Overall, the gain-of-function mouse models indicate that large G4C2 repeat expansions greatly contribute to the pathogenesis of ALS/FTD.

## 5 Potential therapeutic strategies

Understanding the pathogenic mechanism of hexanucleotide repeat expansions in ALS/FTD not only sheds light on the underlying biology of these diseases but also provides multiple potential drug targets for the therapeutic interventions. Various approaches have shown promise as potential treatments for ALS/FTD targeting C9orf72.

### 5.1 Targeting genomic C9orf72 hexanucleotide repeat expansions

The most straight-forward target is the genomic C9orf72 hexanucleotide repeat expansions. CRISPR/Cas9-mediated excision of G4C2 repeat expansions in neurons and mouse models resulted in reduction of RNA foci and DPR inclusions (Meijboom et al., 2022). Since CRISPR/Cas9-mediated genome editing may result in the risk of creating indels (Selvaraj et al., 2018), the combination of CRISPR/Cas9 genome editing and homology-directed repair (HDR) completely repaired the C9orf72 G4C2 repeat expansions to the wild-type repeat size in iPSC cells derived from ALS/FTD patient, and finally abolished pathological phenotypes (Ababneh et al., 2020). The key points for the development of CRISPR/Cas9-based therapeutic strategy should be the efficient and effective delivery, as well as accurate editing of CRISPR/Cas9.

### 5.2 Targeting transcribed RNA

As the accumulation of RNA foci is the pathological hallmark and contributes to the pathology of ALS/FTD, the transcribed r(G4C2)n as a potential drug target for C9orf72-linked ALS/FTD has garnered considerable attention from researchers. One of the therapeutic strategies is degradation of the RNA transcripts using antisense oligonucleotides (ASOs) (Donnelly et al., 2013; Lagier-Tourenne et al., 2013). ASO-mediated degradation of repeat RNA decreased RNA foci and DPR inclusions, as well as ameliorated behavioral deficits (Jiang et al., 2016). Another promising development targeting transcribed r(G4C2)n is that a small molecule, TMPyP4, was characterized to be able to distort the G-quadruplex formation of r(G4C2)8, and ablate the interaction between the G-quadruplex and its binding proteins (Zamiri et al., 2014). Su et al. (2014) further designed and screened small molecules targeting r(G4C2)n to inhibit repeat-associated non-ATG translation and the formation of RNA foci. The findings from their work suggest that small molecules targeting r(G4C2)n hold promise as a potential therapeutic approach for C9orf72-linked ALS/FTD (Su et al., 2014).

### 5.3 Targeting DPR proteins

The toxicity of DPR proteins is the essential part for ALS/FTD pathogenesis, thus the immunotherapeutic approach targeting DPR proteins is another potential therapeutic strategy. In cell and mouse models, the antibodies targeting poly(GA) could reduce poly(GA), poly(GP) and poly(GR) inclusions, improve behavioral deficits, decrease neuroinflammation and neurodegeneration, and increase survival (Zhou et al., 2017, 2020; Nguyen et al., 2020b; Jambeau et al., 2022). On the other hand, it was reported that RAN translation is highly regulated by PKR. The FDA-approved drug, metformin, inhibited PKR, leading to decrease DPR proteins and improve behavior deficits (Zu et al., 2020).

### 5.4 Targeting other downstream mechanisms

Dysfunctional nucleocytoplasmic transport has been characterized as a critical downstream mechanism for ALS/FTD pathogenesis (Prpar Mihevc et al., 2017). KPT-276, the exportin 1 inhibitor, inhibits nuclear export to compensate for disrupted import by the interaction between r(G4C2)n and RanGAP, and suppresses the neurodegeneration in the fly eye (Zhang et al., 2015). Thus, the modulation of nucleocytoplasmic transport is also a potential therapeutic strategy for C9orf72-linked neurodegenerative diseases. ER stress induced by DPR proteins is one of the key downstream pathogenic mechanism. It was reported the inhibitors of ER stress could provide rescue against poly(GA)-induced ER stress and neurotoxicity in neurons (Zhang et al., 2014). Furthermore, pharmacological or genetic suppression of oxidative stress and cellular toxicity induced by poly(GR) decreases DNA damage, indicating reducing oxidative stress is a potential therapeutic strategy for C9orf72-linked ALS/FTD (Lopez-Gonzalez et al., 2016). These advancements mark important steps toward the development of targeted therapies for these debilitating neurodegenerative diseases.

## 6 Conclusion

The hexanucleotide repeat expansions located in the first intron of C9orf72 gene are the most common genetic cause of ALS/FTD. Accumulating research emphasizes the critical role of these hexanucleotide repeat expansions in the pathogenesis of the diseases. The unique sequence of the hexanucleotide, GGGGCC, facilitates the formation of polymorphic secondary structures known as G-quadruplexes, both in the DNA and transcribed RNA. These multimeric G-quadruplexes further promote the formation of RNA foci via phase separation, when the repeat number exceeds a certain threshold. The formation of unusual secondary structures in hexanucleotide repeat expansions greatly impairs the expression of C9orf72 proteins. Furthermore, the accumulation of toxic DPR proteins produced by unconventional translation (i.e., RAN translation) generates inclusions in neuron, which is considered as a distinctive pathognomonic feature of ALS/FTD. Finally, the transcribed G4C2 RNA and its interaction with binding proteins can trigger the formation of RNA granules via phase separation and sequester specific proteins, contributing to the toxicity associated with hexanucleotide repeat expansions.

## Author contributions

YG: Funding acquisition, Writing – original draft, Writing – review and editing. QC: Funding acquisition, Supervision, Writing – original draft, Writing – review and editing.
